# Low-Light Image Enhancement Using Photometric Alignment with Hierarchy Pyramid Network

**DOI:** 10.3390/s22186799

**Published:** 2022-09-08

**Authors:** Jing Ye, Xintao Chen, Changzhen Qiu, Zhiyong Zhang

**Affiliations:** School of Electronics and Communication Engineering, Sun Yat-sen University, Shenzhen 518107, China

**Keywords:** low-light image enhancement, coarse-to-fine, photometric alignment

## Abstract

Low-light image enhancement can effectively assist high-level vision tasks that often fail in poor illumination conditions. Most previous data-driven methods, however, implemented enhancement directly from severely degraded low-light images that may provide undesirable enhancement results, including blurred detail, intensive noise, and distorted color. In this paper, inspired by a coarse-to-fine strategy, we propose an end-to-end image-level alignment with pixel-wise perceptual information enhancement pipeline for low-light image enhancement. A coarse adaptive global photometric alignment sub-network is constructed to reduce style differences, which facilitates improving illumination and revealing under-exposure area information. After the learned aligned image, a hierarchy pyramid enhancement sub-network is used to optimize image quality, which helps to remove amplified noise and enhance the local detail of low-light images. We also propose a multi-residual cascade attention block (MRCAB) that involves channel split and concatenation strategy, polarized self-attention mechanism, which leads to high-resolution reconstruction images in perceptual quality. Extensive experiments have demonstrated the effectiveness of our method on various datasets and significantly outperformed other state-of-the-art methods in detail and color reproduction.

## 1. Introduction

The presence of low-light images in high-level vision tasks is inevitable, and image enhancement has a significant effect on performance improvement. However, low-light images captured in dim environments and back-lit conditions often suffer from severe degradation, including poor visibility, intensive noise, and biased color. Although long exposure shooting allows the photosensitive sensor to receive more light, which can improve the illumination of the images to a certain extent, it is impractical for real-time demanding tasks such as autonomous driving and target tracking. Due to the limitations of the camera device sensor hardware, the use of algorithms to mitigate low-light image degradation has become a research hotspot. Low-light image enhancement is mainly aimed at improving the visibility of images, removing noise, and enhancing contrast to achieve pleasant human perception effects. In the past decades, a large number of low-light image enhancement algorithms have been proposed, which can be broadly classified into the following three types: global adjustment methods, Retinex-based methods, and learning-based methods.

The main global adjustment methods include histogram equalization (HE) and gamma correction (GC). Early HE methods [1,2] enhanced the contrast by stretching the dynamic range of the images according to the histogram, and some variants of this method have been developed [3,4,5]. Still, the strict requirement of histogram uniform distribution severely limits the enhancement performance of such methods. The GC methods [6] adjust the values of the pixel points through an exponential function, which works on individual pixel points and ignores the relationship between adjacent pixel points, leading inevitably to over-exposure and noise amplification in the enhanced image.

Based on the traditional Retinex theory [7], the initial Retinex-based methods [8,9,10] estimated the illuminance and reflectance maps of low-light images and enhanced these two components separately before fusing the output results. In recent years, several improved Retinex methods [11,12,13,14] also have been proposed to better decompose the illuminance and reflectance components by imposing prior knowledge. In [15,16], an estimated noise map was integrated into robust Retinex model to remove noise and achieve low-light enhancement.

Due to the powerful inference capabilities of machine learning techniques, learning-based methods started to develop rapidly in the field of low-light image enhancement. Benefiting from the availability of real-world paired low-/normal-light image datasets, massive methods [17,18,19,20,21,22,23] combine Retinex theory with deep networks, learning to estimate potential components, adjust the illumination map, and alleviate the degradations of reflectance layer for achieving natural low-light image enhancement. Yang et al. [24] proposed a band representation-based semi-supervised method to restore signal fidelity and perceptual quality. In [25,26,27], deep networks are constructed to generate and discriminate the high-visual-quality images,; these methods release the limitations of paired datasets, effectively avoid model overfitting, and improve the generalization performance on real datasets.

**Figure 1:**
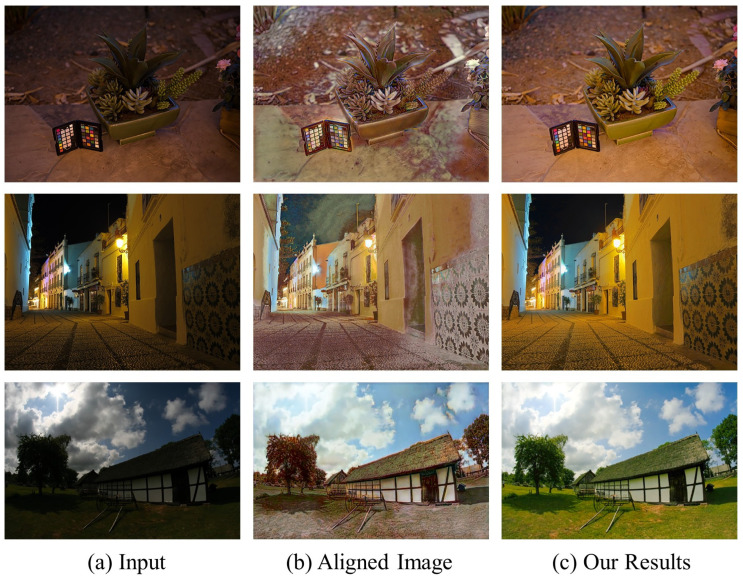
Examples of low-light image enhancement using our method. (**a**) Input low-light images. (**b**) The aligned images resulting from adaptive global photometric alignment improve the contrast and reveal underexposed regions. (**c**) The final results display rich detail and vibrant color.

Although existing learning-based methods can achieve good performance in some cases, there are still some general issues. Most of the models generate underexposed and overexposed images, lose texture and detail information during enhancement, and unpaired data training often fails to cope with distorted color and amplified noise. Simultaneously improving illumination, denoising, and restoring natural color is a non-trivial problem [28]. To address these challenges, this paper proposes an end-to-end image-level alignment with pixel-wise perceptual information enhancement pipeline for low-light image enhancement. The key insight is to minimize the style differences [29] between input low-light images and target images using an image-level alignment strategy in the coarse stage, to recover visually pleasing results at the refinement stage. Specifically, different from existing global photometric alignment methods [29] that require complicated histogram matching and gamma correction of the source domain image set, we elaborately devise a style-consistency loss to facilitate supervised learning of a global photometric alignment sub-network, which is beneficial for the adaptive style transfer of low-light images. As shown in Figure 1b, we minimize the style differences [29] (e.g., exposure, contrast, lighting, object shape, and surface textures). In the refinement stage, we develop a hierarchical pyramid enhancement sub-network to remove the amplified noise, optimize the local detail, and restore the vivid color of images; an example is given in Figure 1c. Additionally, to avoid generating artifacts and other degradations, we also design a multi-residual cascaded attention block (MRCAB), which facilitates multi-scale feature extraction and high-resolution reconstruction. The main contributions are summarized as follows:

- We propose a novel coarse-to-fine adaptive low-light image enhancement network (CFANet) that seamlessly combines coarse global photometric alignment with finer perceptual information promotion. The coarse-to-fine pipeline is trained in a data-driven manner within a unified framework to avoid error accumulation.
- The built MRCAB is embedded into a hierarchy pyramid network, which can change the perceptual fields and highlight notable features for each network layer. Furthermore, the polarized self-attention mechanism of the block can preserve high-resolution information to achieve better enhancement performance.
- Experiments show that our method can generalize well across different real low-light datasets. Specially, we restore less noise normal-light images with rich detail and vivid colors compared to other low-light enhancement methods.

## 2. Related Work

### 2.1. Traditional Methods

Traditional methods mainly review global adjustment methods and Retinex-based methods. The classical global HE method [1,2] implements nonlinear stretching to enhance image contrast and reveal the content of underexposed areas, but it may cause overexposure and drowning of detail by over-transforming the saturated regions. To cope with this problem, in the local HE method [3], the global histogram is sliced into multiple sub-histograms and the enhancement operations are performed separately in different regions, which helps to improve the performance of low-light image enhancement flexibly. However, these methods increase the computational complexity to some extent. Therefore, the parametric-oriented HE methods [4,5] attempt to reduce the enhancement complexity by optimizing the transformation process to a uniform function that maps the low-light images to the output results. Huang et al. [6] improved the contrast of images by gamma correction of luminance pixels. However, the above methods are not designed for low-light image enhancement especially, and the enhancement results often show hard noise and unnatural results.

Single-scale Retinex [8] is the first practical application of Retinex theory to image processing, and it is found that the surround formation produces the best enhancement results. Single-scale Retinex was extended to Multi-scale Retinex [9] to achieve both color and luminance recovery. Some studies [11,12,13] decomposed the illumination and reflectance components by using artificially designed priors, ignoring the degradations of the reflectance layer, which may lead to strong noise in the output. On the other hand, a noise prior was added to constructing a robust Retinex model for enhancing low-illumination images in [15,16]. The prior design of the above methods is too complex and cannot satisfy the adaptive enhancement of the real low-light images.

### 2.2. Learning-Based Methods

Learning-based methods have achieved extraordinary results in several vision domains. Loreet al. [30] was the first to explore the application of deep learning in the field of low-light image enhancement and proposed the deep autoencoder-based method (LLNet), which obtained impressive enhancement results. In [17,31], LOL and SID real low-/normal-light pairs were proposed to accelerate the development of learning-based low-light enhancement methods. MBLLEN [32] uses multi-branch sub-networks to enhance the different layer inputs separately, then outputs the fusion results. Zhang et al. [18] constructed three sub-networks for decomposition, adjustment of illumination, and recovery of reflectance components, respectively. In [22], KinD++ was proposed to mitigate visual defects (non-uniform spots and over-smoothing) left in KinD [18]. Lu et al. [33] proposed slight and heavy adaptive attention mechanisms for low-light image enhancement with different degrees of degradations. Li et al. [34] used a luminance-aware pyramidal structure to enhance the local and global features of the low-light images. These works focus on enhancing severely degraded low-light images directly via improving the network structure. However, simultaneously boosting illumination, removing noise, and restoring detail can lead to undesirable results. Some later methods adopted more special deep networks than the previous works. Li et al. [35,36] used the deep curve estimation to achieve impressive results. Jiang et al. [25] first introduced an unpaired learning strategy to build a new pipeline EnlightenGAN, which greatly improved the generalization performance of the model. Pan et al. [26] proposed a multi-module cascade generative network and adaptive multi-scale discriminative network. However, unsupervised methods lack the guidance of paired data and need to be further improved in terms of image fidelity and color recovery ability.

Comparatively, this paper enhances low-light images in a coarse-to-fine manner. Inspired by the image-level domain shift strategy [29], an adaptive global photometric alignment sub-network is used to shift the style of severe degraded low-light images, including exposure, contrast, and texture, with the ability to explore the content in underexposed regions. In the optimization stage, local detail, and color of the aligned image are further enhanced to remove the amplified noise and generate visually pleasurable images.

## 3. Coarse-to-Fine Enhancement Pipeline

### 3.1. Motivation

Deep methods can effectively enhance the quality of low-light images. In general, performing enhancement tasks directly from severely degraded low-light images usually yield undesirable results. In other words, simultaneously enhancing illumination, removing noise terms, and restoring vivid color would also be a very difficult task.

Is it possible to perform low-light image enhancement progressively? The methods in [33,37] support this view. However, the former requires stepwise training of two independent networks, which is prone to the accumulation of model errors. Additionally, the latter lacks the guidance of paired data, and there are amplified noise and distorted colors in the recovered results. Based on the above observations, it is feasible to effectively enhance low-light images in a coarse-to-fine manner, and the enhancement work is facilitated by obtaining an intermediate image from the input image that is close to the target image in terms of brightness, contrast, and content. This is essentially different from the coarse-to-fine network framework used in [34,38] for extracting multi-scale features. Furthermore, a recently proposed domain shifts problem [29] inspired us. In their work, the image-level alignment is used to decrease the domain shifts. Based on the success of the semantic segmentation task, the idea of estimating photometric aligned images motivated us to extend it to style transformation in low-light image enhancement. However, performing classic histogram matching on color channels and lightness gamma correction on the light channel ignores the association between the channels and cannot accommodate diverse low-light image enhancement.

Based on the above insights, our CFANet attempts to enhance low-light images at the image-level and pixel-wise in a coarse-to-fine fashion, adaptive style transfer of low-light images is implemented using a deep model, which better simulates the enhancement of low-light images and works effectively within a unified network framework. Particularly, the intermediate style consistency loss can better boost brightness and explore content in underexposed areas. This unique design allows CFANet to overcome the problem of independent channel adjustment. Hence, it trades off well between style difference and content preservation.

### 3.2. CFANet

**Figure 2:**
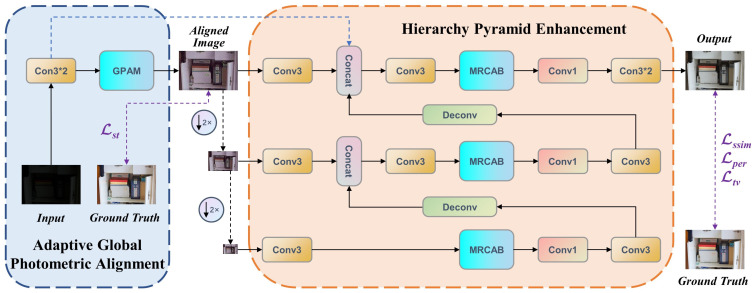
An illustration of the proposed CFANet. It contains an adaptive global photometric alignment sub-network for style transformation, and a hierarchy pyramid enhancement sub-network for optimization of the image quality. We build the GPAM with the basic U-net structure. The proposed MRCABs are inserted in hierarchy pyramid architecture to extract multi-scale features in a wider range, suppressing artifacts and color distortion more efficiently. The low-light images are mapped to the output in a coarse-to-fine manner.

Figure 2 shows the overall architecture of CFANet, which can be divided into two sub-networks. The coarse adaptive global photometric alignment sub-network learns the style transformation of low-light images, and the finer hierarchy pyramid enhancement sub-network uses multi-residual cascade attention blocks (MRCABs) to further optimize the aligned images. We described the two sub-networks and MRCAB in detail below.

#### 3.2.1. Network Architecture

In the coarse stage, the input low-light images are fed into an adaptive global photometric alignment sub-network that is designed to decrease style differences with the supervision of style consistency loss $L_{st}$ (see Section 3.3). Therefore, given a collection of *M* image pairs ${\left\{I_{in}^{m},I_{gt}^{m}\right\}}_{m=1}^{M}$, we aim to solve the following problem:

(1) $$\gamma^{*}=arg\min_{\gamma }\frac{1}{M}\sum_{m=1}^{M}L_{st}\left(E_{AGPA_{\gamma }}\left(I_{in}^{m}\right),I_{gt}^{m}\right),$$

where $I_{in}^{m}$ and $I_{gt}^{m}$ denote the input image and ground truth, respectively. γ is the parameter set and $E_{AGPA}\left(\cdot \right)$ represents the adaptive global photometric alignment sub-network. Here, $L_{st}\left(\cdot \right)$ is adopted to minimize the style difference between the aligned image and ground truth. The coarse network consists of two 3 × 3 convolutional layers and a global photometric alignment module (GPAM), as shown in Figure 2. Two convolutional layers in the front-end of the network are first used to explore shallow features of low-light images. After that, we build the GPAM with the basic U-net [39] structure, [25,36] also demonstrated the effectiveness of the U-net in low-light image enhancement. Thanks to the skip connections between the downsampling and upsampling layers of the GPAM, the sub-network can preserve the structure information of the original images while enhancing the brightness, contrast, and exploring the content of underexposed regions during the style transformation. To facilitate intermediate supervision, we output an aligned image by the last convolution layer. Our design gears adaptive global photometric alignment sub-network to embed the input low-light images into the feature space of aligned images, allowing the subsequent hierarchy pyramid enhancement sub-network to pay more attention to optimization tasks.

Although the aligned images from the adaptive global photometric alignment sub-network are close to the target images in terms of luminance, contrast, and surface texture. However, as can be observed in Figure 1b, there are still color distortions, artifacts, and amplified noise. In the refinement stage, the hierarchy pyramid enhancement sub-network focuses on optimizing the above problems. Essentially, this sub-network also enhances the features in a coarse-to-fine strategy. The aligned images are used as input images of different resolutions after downsampling. Although different branches consist of MRCAB with the same structure, the multi-scale network can enhance global and local features from the bottom up, respectively. Furthermore, to avoid the effect of detail information loss caused by over-convolution, after all global features are pooled to the top branch by the deconvolution operation, a skip connection is established to share shallow features to improve the refinement features and generate the final results.

In particular, it is important to note that our CFANet is implemented in a data-driven manner within a unified framework, which is beneficial for decreasing error accumulation and restoring desirable normal-light images.

#### 3.2.2. Multi-Residual Cascade Attention Block (MRCAB)

**Figure 3:**
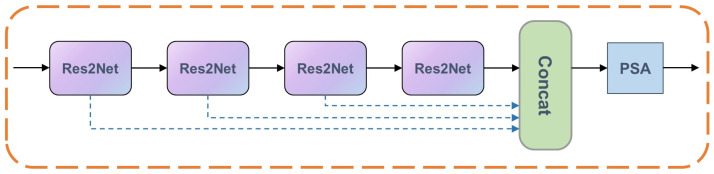
An illustration of our proposed multi-residual cascade attention block(MRCAB), which is the crucial component of the CFANet. It consists of four cascaded Res2Net and a polarized self-attention (PSA) block.

Both photometric alignment and perceptual quality improvement in our task are spatially varying problems. Though the hierarchy pyramid architecture can explore features at different scales, it is not enough for image quality optimization tasks. Typical low-light image enhancement networks are prone to artifacts and unnatural color, and we find that these problems can be significantly remedied by changing the perceptual field of the network, highlighting and suppressing features. To achieve this goal, we elaborately devise the MRCAB, which consists of four cascaded Res2Net [40] and a polarized self-attention (PSA) block [41] (see Section 5 for a detailed description of the cascade number settings of Res2Net), as shown in Figure 3. The Res2Net adopts channel split and concatenation strategy to form different receptive fields to effectively extract multi-scale features. Rather than using the SE block [42], we choose a PSA block that is more adapted to pixel-wise regression, the attention mechanism simultaneously maintains high resolution in both channel and space, and achieves nonlinear enhancement of high-resolution information. Specifically, skip connections within MRCAB allow for more efficient utilization and propagation of hierarchical feature information. We would like to highlight that MRCAB is the essential component in our network, it suppresses artifacts and color distortion that may be caused by the co-existence of other degradations of oversaturation and noise.

### 3.3. Loss Function

To enable supervised learning in the coarse-to-fine pipeline, our proposed loss function consists of the following four parts.

**Style consistency loss.** To reduce the style difference between the aligned image and the ground truth, boost the brightness and preserve the detail of the original image. Specifically, we provide intermediate supervision at the end of the adaptive global photometric alignment sub-network. The style constancy loss $L_{st}$ can be expressed as:

(2) $$L_{st}=\sum_{c\in \xi }^{}\left|J_{a}^{c}-J_{g}^{c}\right|,\xi =\left\{R,G,B\right\},$$

where $J_{a}^{c}$, $J_{g}^{c}$ denote the intensity value of the aligned image and the ground truth in channel *c*, respectively.

**Structure similarity loss.** Since MAE and MSE losses ignore the correlation between the long-distance of pixel points, this makes it difficult to overcome structural distortions such as artifacts and blurring. Therefore, we introduce the structure similarity loss to enhance the recovery quality of low-light images. The structure similarity loss $L_{ssim}$ is defined as:

(3) $$L_{ssim}=1-\frac{2\mu_{x}\mu_{y}+c_{1}}{\mu_{x}^{2}+\mu_{y}^{2}+c_{1}}\cdot \frac{2\sigma_{xy}\;+c_{2}}{\sigma_{x}^{2}+\sigma_{y}^{2}+c_{2}},$$

where $\mu_{x}$, $\mu_{y}$ represent the pixel average value of *x*, *y* images, respectively. $\sigma_{x}^{2}$ and $\sigma_{y}^{2}$ are variances, the covariance is represented as $\sigma_{xy}$. To avoid the denominator being zero, $c_{1}$, $c_{2}$ are set to 0.0001 and 0.0009 in our work, following the same setting in Wang et al. [43].

**Perceptual loss.** To facilitate the enhancement of image perceptual information, we fed the enhanced image and the ground truth into the pre-trained VGG-19 network to measure the difference between the corresponding feature maps. The perceptual loss $L_{per}$ can be expressed as:

(4) $$L_{per}=\frac{1}{C_{n}H_{n}W_{n}}{∥\phi_{n}\left(E\left(I_{in}\right)\right)-\phi_{n}\left(I_{gt}\right)∥}_{2}^{2},$$

where $E\left(\cdot \right)$ represents our CFANet. $\phi_{n}\left(\cdot \right)$ is the feature map of the *n*-th convolutional layer in VGG-19 model. $C_{n}$, $H_{n}$, $W_{n}$ denote the dimensions of the corresponding feature maps, respectively.

**Total variation loss.** To remove noise and improve the visual effect of the images, total variation loss is introduced to limit the gradient of the images. The total variation loss $L_{tv}$ is written as:

(5) $$L_{tv}=\sum_{i=1}^{H}\sum_{j=1}^{W}\sqrt{\left(p_{i,j}-p_{i+1,j}\right)\left(p_{i,j}-p_{i,j+1}\right)},$$

where *p* represents the intensity value at pixel point index $\left(i,j\right)$.

**Total loss.** The total loss function is:

(6) $$L_{total}=\lambda_{st}L_{st}+L_{ssim}+\lambda_{per}L_{per}+\lambda_{tv}L_{tv}.$$

We set the loss weights of $\lambda_{st}$, $\lambda_{per}$, and $\lambda_{tv}$ to 0.1, 0.2, and 0.01, respectively, in our experiments.

## 4. Experiments

### 4.1. Datasets and Evaluate Metrics

We train our CFANet and other state-of-the-art methods using LOL [17] and SID [31] datasets. The LOL dataset consists of 500 real scenes image pairs and 1000 synthetic image pairs, the SID dataset in RAW format is converted to sRGB format for training. Additionally, we also evaluated on LIME [12], MEF [44], NPE [10], DICM [5], VV [45] datasets to demonstrate the effectiveness and generality of our approach. We adopt the commonly used PSNR, SSIM [46], and NIQE [47] metrics for evaluation.

### 4.2. Experimental Settings

The proposed CFANet is designed based on the Pytorch framework. We randomly crop 256 × 256 patches for training on NVIDIA RTX 3090 GPU, all these patches are transformed by randomly flipping and rotations of $90^{\circ }$, $180^{\circ }$, $270^{\circ }$. The network is trained on the LOL and SID datasets for 400,300 epochs, respectively. The former has an initial learning rate of $10^{-4}$, which is halved at 200 epochs; and the latter has an initial learning rate of $10^{-3}$, which is halved at 150 epochs. The mini-batch is set to 8. We train our network using Adam optimizer with $\beta_{1}=0.9$; $\beta_{2}=0.99$.

### 4.3. Enhancement Results

To comprehensively evaluate the low-light image enhancement performance of CFANet, we performed quantitative evaluations on LOL, SID, LIME, MEF, NPE, DICM, VV datasets, and qualitative comparisons on datasets besides SID.

#### 4.3.1. Quantitative Evaluation

We choose recent light enhancement networks to evaluate the performance of LOL synthetic and real datasets, which is consistent with the evaluation approach in [21,24], including BIMEF [48], CRM [49], DHECE [50], Dong [51], EFF [52], LIME [12], MF [11], MBLLEN [32], JED [16], SRIE [13], RRM [15], DRD [17], DeepUPE [53], SCIE [54], KinD [18], EnlightenGAN [25], RetinexNet [21], KinD++ [22], and DRBN [24]. As shown in Table 1, we found both on synthetic and real LOL datasets that our method achieves the best results in both PSNR and SSIM metrics compared to the state-of-the-art methods. The results suggest that CFANet is effective and particularly well-suited for low-light image enhancement tasks.

Since linear RAW data is significantly different from nonlinear sRGB data, the model trained in RAW format cannot be adapted to enhance sRGB images, and the image format acquired by photographic devices is usually sRGB [55]. Therefore, this paper only compares with networks trained on the SID dataset in sRGB format, including DSLR [56], LIME [12], SCIE [54], DeepUPE [53], and LRD [55]. For the test results of the SID dataset in Table 2, we found that our network achieved the best results in the PSNR metric and comparable results in the SSIM metric, which shows the superiority of our coarse-to-fine strategy and losses.

We evaluated the proposed CFANet and nine representative methods on several real datasets LIME, MEF, NPE, DICM, and VV. Table 3 shows the NIQE metric test results, no single method can achieve the best score on all datasets, but our method performs the best on NPE and DICM datasets, and otherwise still maintains a good score on other datasets. The comparisons in real datasets strongly suggest the effectiveness and generality of our proposed network.

#### 4.3.2. Qualitative Evaluation

**Figure 4:**
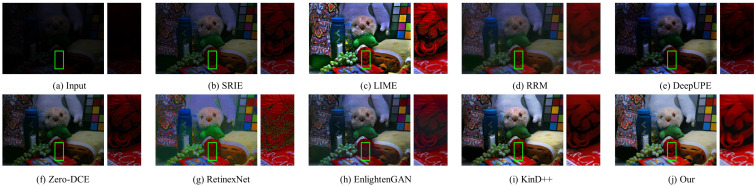
Visual comparison of low-light image enhancement on an image from the LOL dataset.

**Figure 5:**
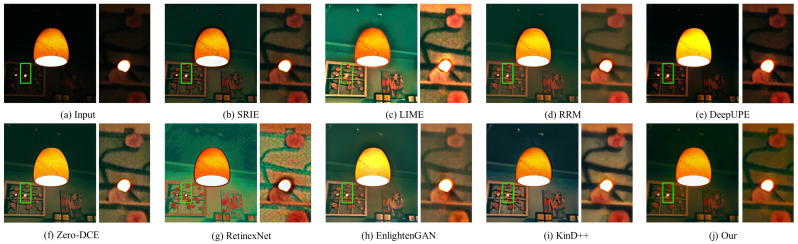
Visual comparison of low-light image enhancement on an image from the LIME dataset.

**Figure 6:**
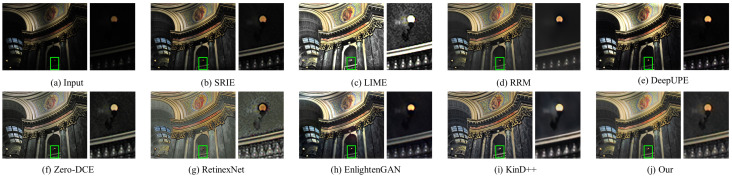
Visual comparison of low-light image enhancement on an image from the MEF dataset.

**Figure 7:**
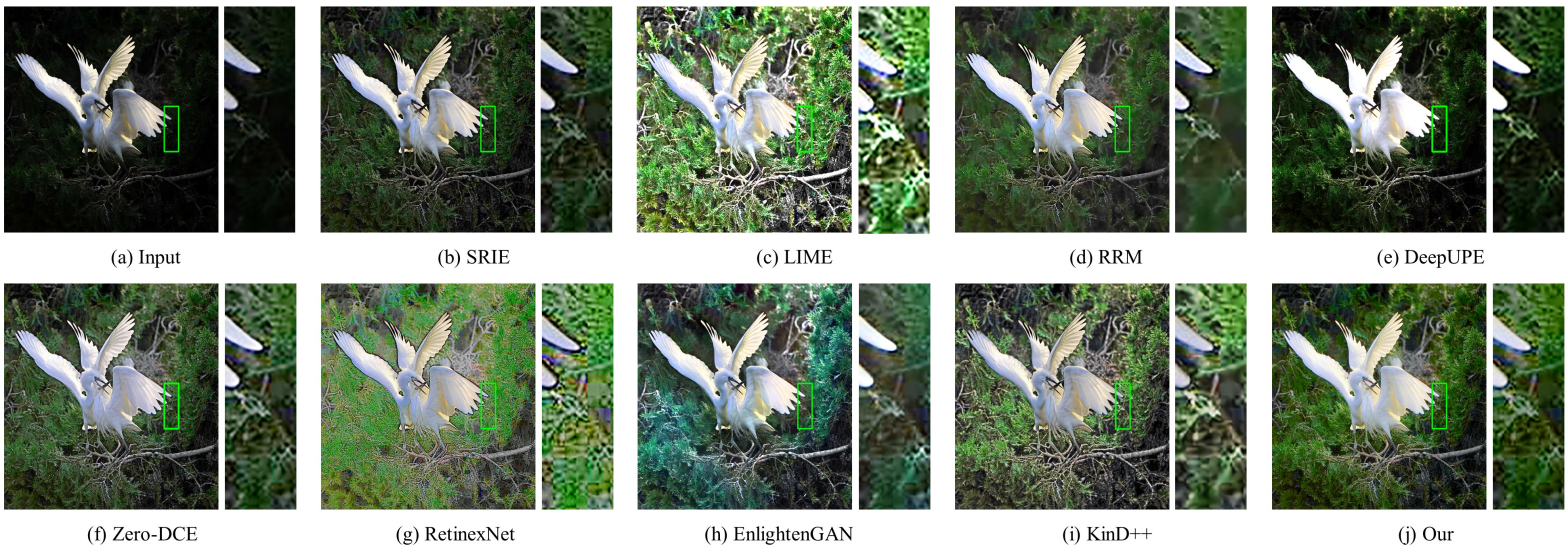
Visual comparison of low-light image enhancement on an image from the NPE dataset.

**Figure 8:**
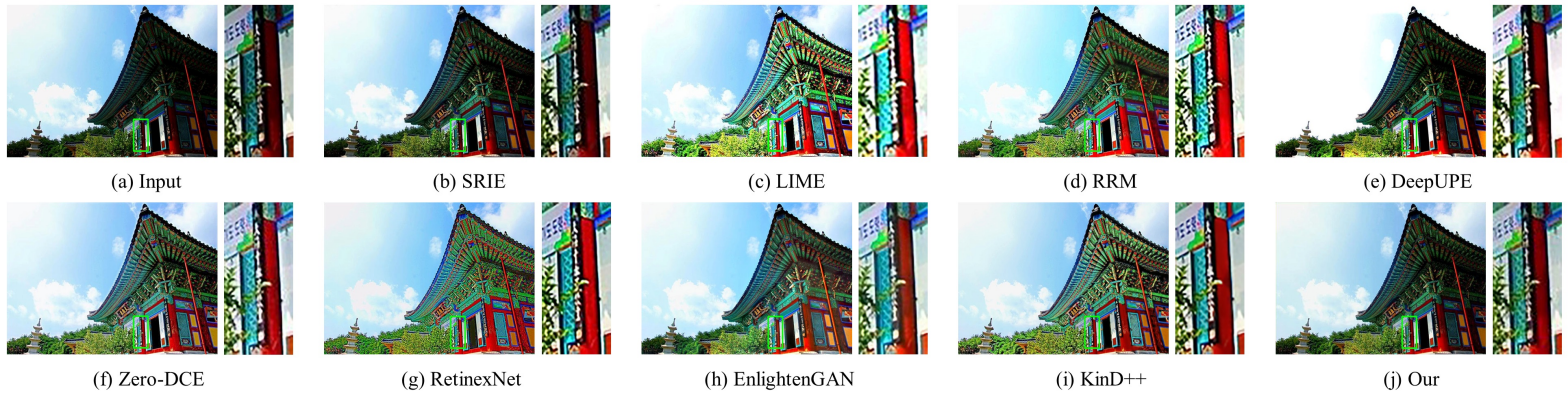
Visual comparison of low-light image enhancement on an image from the DICM dataset.

**Figure 9:**
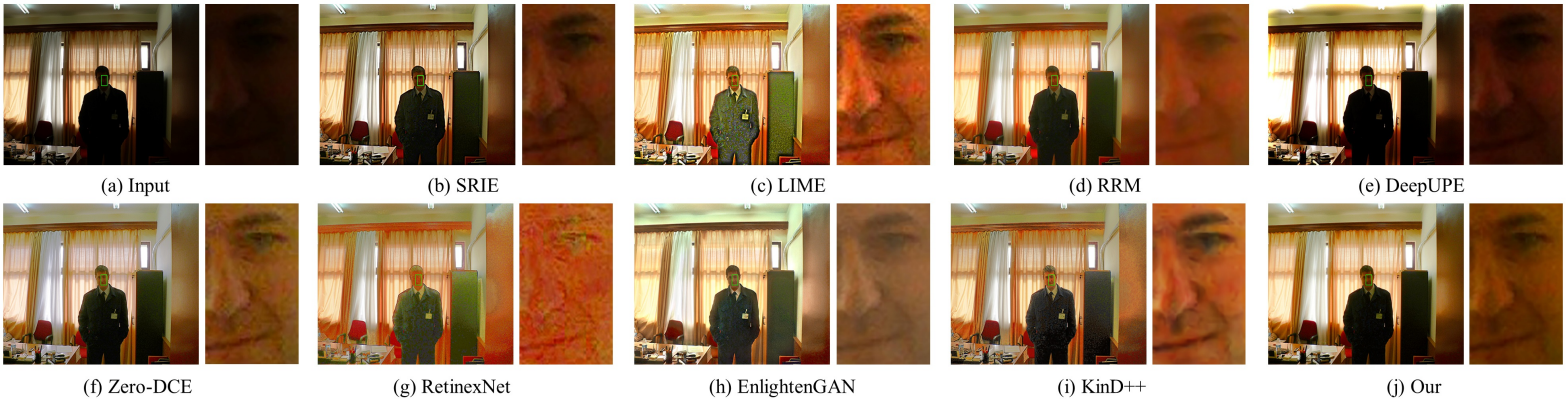
Visual comparison of low-light image enhancement on an image from the VV dataset.

In this part, the results of three traditional methods and five deep learning methods in Figure 4, Figure 5, Figure 6, Figure 7, Figure 8 and Figure 9 are described in detail in comparison with our network in terms of visual effects. We found that SRIE produced underexposed enhancement results in most cases (e.g., Figure 4, Figure 6, Figure 8 and Figure 9) and less improvement for image contrast. LIME generated several overexposed regions in Figure 6, Figure 7 and Figure 8, and the work adopted the denoising mechanism as post-processing still caused strong noise and artifacts. To effectively reduce the effect of noise, RRM improves the robustness by estimating the noise map in the model, but over smoothing the image causes blurring of the main structures and loss of image detail information in Figure 4, Figure 5, Figure 7 and Figure 9. Figure 4, Figure 5, Figure 6 and Figure 9 show that the low-light enhancement performance of DeepUPE is weak, producing a large number of unexposed areas. Early RetinexNet performed poorly in terms of enhancement performance, with significant noise and artifacts in all enhanced images. The unsupervised methods Zero-DCE and EnlightenGAN are trained on unpaired data, they restore relatively impressive results on different datasets, but also suffer from color distortion (e.g., Figure 5 and Figure 7) and fail to cope with extremely dark regions (e.g., Figure 4). KinD++ overcame the visual defects of excessive smoothing and uneven brightness to a certain extent by improving the KinD method; however, we found that there are still problems of unclear image detail and low contrast in Figure 4 and Figure 7.

In comparison with the above results, we restored normal-light images of good visual quality in all enhancement experiments. Thanks to the particular frame design of CFANet, our method can explore the content of underexposed regions using the adaptive global light alignment module while maintaining high resolution. In particular, as shown in Figure 4, Figure 5 and Figure 7, beneficial from the coarse-to-fine strategy, the images processed through our network exhibit stunning colors and excellent contrast, with clear detail and good illumination for a pleasant visual effect. The visual comparison in various cases indicates the superiority and generalization of our approach.

Though our method achieves promising results in most cases, but we also found that the model may show fragile performance in extreme darkness, such as the artifacts in the face region of Figure 9, which is degradation caused by over-smoothing to avoid noise. To cope with this limitation, we plan to implicitly incorporate the denoising process into our model to mitigate this problem in the future.

## 5. Ablation Study

In this section, we present an ablation study to demonstrate the effectiveness of the main components in CFANet and losses, which was performed on the LOL dataset.

**Effectiveness of network architecture.** As shown in Table 4, in the absence of a global photometric alignment module, the Res2Net module performs slightly better than the ResNet module, and the performance is further improved by adding the PSA mechanism, while our proposed CFANet, which includes the global light alignment module, achieves the best scores on both PSNR and SSIM metrics. The above quantitative results demonstrated the effectiveness of our network components.

**Figure 10:**
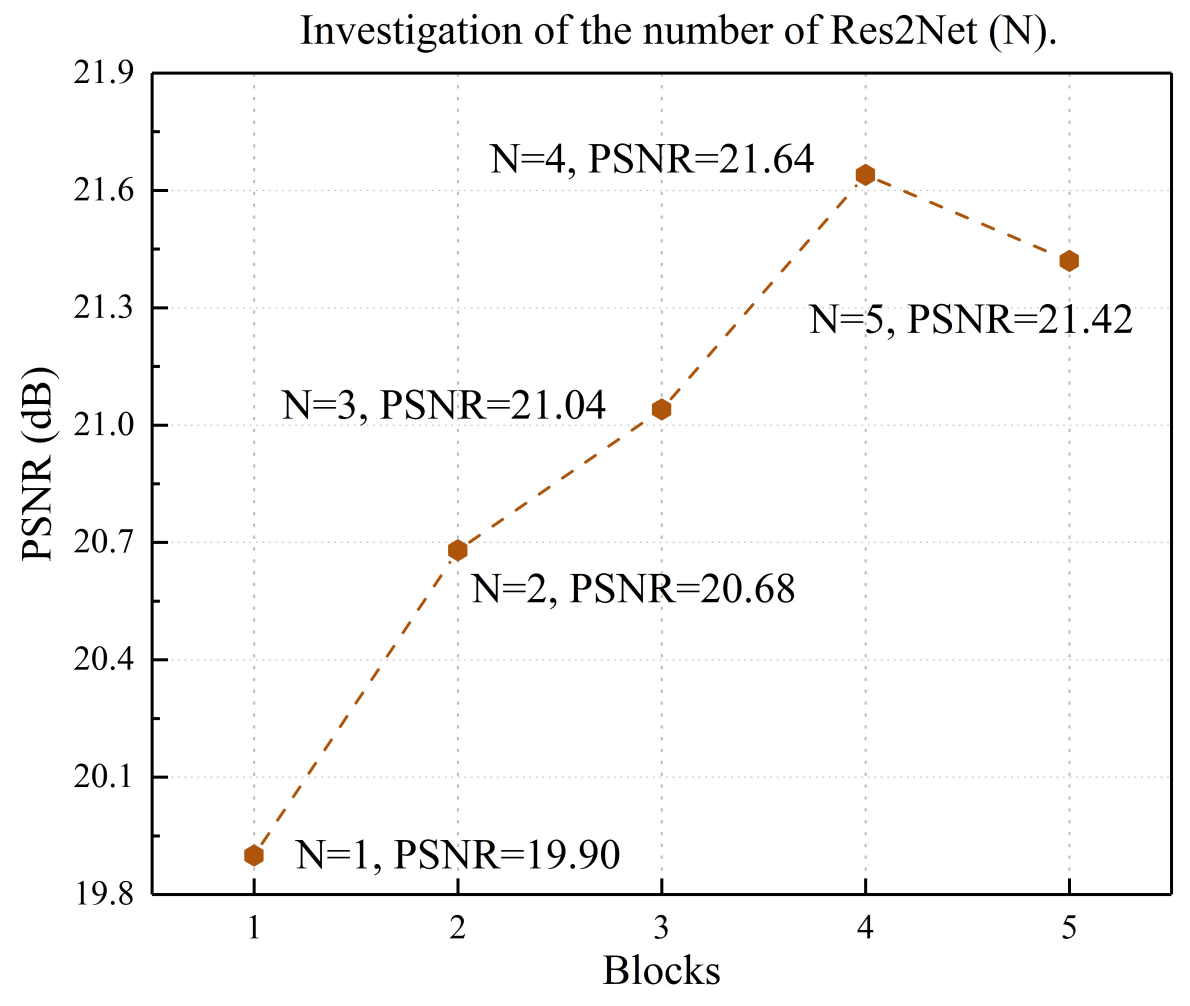
Investigate the effect of Res2Net block number for enhancement performance on the LOL dataset.

To investigate the effect of the number of Res2Net blocks in MRCAB on low-light image enhancement, we set different numbers of blocks to train the model. As we can see in Figure 10, with the increase of Res2Net blocks, the network gradually improves the PSNR. When the number of blocks exceeds four, the benefit of improving PSNR disappears, and the network is prone to overfitting results. We found that the optimal setting for the number of blocks is N = 4.

**Effectiveness of losses.** We verify the validity of each loss function by adding them step by step. In Table 5, removing arbitrary losses degrades the network performance. The combination of style consistency loss, structural similarity loss, perceptual loss, and total variance loss achieves the best performance, which also indicates that the intermediate style consistency loss is effective for our network.

## 6. Conclusions

In this paper, we have presented a novel coarse-to-fine adaptive low-light image enhancement pipeline that seamlessly combined coarse global photometric alignment with finer perceptual information promotion. With the coarse adaptive global photometric alignment subnet, the difference in style between low-light and normal-light images is effectively reduced, facilitating improved illumination and revealing information in underexposed areas. Moreover, the proposed multi-residual cascade attention block (MRCAB) is designed to be embedded in the backbone network, which allows CFANet to avoid degradations and maintain high resolution. Compared to other low-light image enhancement algorithms, our proposed CFANet achieves significant improvements in PSNR and SSIM, and restores suitable illumination, rich detail information, and vivid colors. Extensive experiments on widely used low-light image datasets have demonstrated the effectiveness and generality of our method.

Our method can effectively mitigate the detail blur of static images. In general, real-world low-light images usually have the problem of image blur caused by fast target movement and camera shake [57], we will explore solutions for the joint task of low-light image enhancement and deblurring in future work.

## Figures and Tables

**Table 1:** Quantitative evaluation on LOL synthetic and real dataset, in terms of PSNR and SSIM. The best results are in bold.

Method	LOL-Syn		LOL-Real	
	PSNR↑	SSIM↑	PSNR↑	SSIM↑
BIMEF	17.20	0.7172	17.85	0.6526
CRM	18.91	0.7864	19.65	0.6623
DHECE	17.75	0.7800	14.64	0.4450
Dong	16.90	0.7487	17.26	0.5270
EFF	17.20	0.7127	17.85	0.6526
LIME	16.88	0.7762	15.24	0.4702
MF	17.50	0.7514	18.73	0.5590
MBLLEN	17.07	0.7301	17.86	0.7247
JED	17.48	0.7444	17.33	0.6654
SRIE	14.50	0.6163	17.34	0.6859
RRM	17.15	0.7277	17.33	0.5144
DRD	17.13	0.7978	15.47	0.5672
DeepUPE	15.08	0.6225	13.27	0.4521
SCIE	18.50	0.7631	19.40	0.6906
KinD	17.84	0.7971	20.73	0.8103
EnlightenGAN	16.57	0.7338	18.23	0.6165
RetinexNet	22.05	0.9054	20.06	0.8158
KinD++	17.69	0.8334	21.30	0.8226
DRBN	23.22	0.9275	20.29	0.8310
Our	**24.62 **	**0.9314**	**21.64**	**0.8481**

**Table 2:** Quantitative evaluation on SID dataset, in terms of PSNR and SSIM. The best results are in bold.

Method	PSNR↑	SSIM↑
DSLR	17.25	0.4229
LIME	17.76	0.3506
SCIE	21.16	0.6398
DeepUPE	21.55	0.6531
LRD	22.13	**0.7172**
Our	**22.60**	0.6728

**Table 3:** Quantitative evaluation with NIQE metric on LIME, MEF, NPE, DICM, and VV datasets. The best results are in bold.

Method	NIQE↓				
	LIME	MEF	NPE	DICM	VV
SRIE	3.3481	3.1601	3.6930	3.4161	3.0015
LIME	3.3176	**2.9363**	3.9679	3.5289	**2.4221**
RRM	4.1056	4.3742	4.3785	3.9799	4.3785
MBLLEN	4.1445	4.1969	4.2200	4.0426	4.0631
KinD	4.5086	3.3126	3.7476	3.8037	2.9148
DeepUPE	3.6233	3.4051	4.0390	3.9296	3.1807
RetinexNet	4.0272	3.9265	4.1013	4.1775	2.5792
EnlightenGAN	**3.1880**	2.9440	3.6775	3.3632	2.5875
KinD++	4.3394	3.3082	3.8462	3.5727	2.5974
Our	3.6706	2.9956	**3.6124**	**3.3186**	3.1158

**Table 4:** Ablation results of network structure on LOL dataset. The best results are in bold.

Metric	Module			
	ResNet	Res2Net	Res2Net + PSA	CFANet
PSNR	19.72	19.94	20.83	**21.64**
SSIM	0.8037	0.8078	0.8143	**0.8481**

**Table 5:** Ablation results of loss function on LOL dataset. The best results are in bold.

Loss Configuration	PSNR	SSIM
1. with $L_{ssim}$, w/o $L_{tv}$, w/o $L_{per}$, w/o $L_{st}$	20.19	0.8225
2. with $L_{ssim}$, with $L_{tv}$, w/o $L_{per}$, w/o $L_{st}$	21.43	0.8341
3. with $L_{ssim}$, with $L_{tv}$, with $L_{per}$, w/o $L_{st}$	21.40	0.8367
4. default configuration	**21.64**	**0.8481**

## Data Availability

The dataset and the code of the comparison method used in this paper are publicly available on github.
